# Crystal structure of (*E*)-*N*-{2-[2-(3-chloro­benzyl­idene)hydrazin­yl]-2-oxoeth­yl}-4-methyl­benzene­sulfonamide monohydrate

**DOI:** 10.1107/S2056989015008506

**Published:** 2015-05-09

**Authors:** H. Purandara, Sabine Foro, B. Thimme Gowda

**Affiliations:** a Department of Chemistry, Mangalore University, Mangalagangotri 574 199, Mangalore, India; b Institute of Materials Science, Darmstadt University of Technology, Alarich-Weiss-Strasse 2, D-64287 Darmstadt, Germany; c Bangalore University, Jnanabharati, Bangalore 560 056, India

**Keywords:** crystal structure, synthesis, aryl­sulfonyl glycinyl hydrazone, hydrogen bonding

## Abstract

The mol­ecule of the title compound, C_16_H_16_ClN_3_O_3_S·H_2_O, is L-shaped being bent at the S atom; the S—N—C—C torsion angle is 132.0 (3)°. The central part of the mol­ecule, C—C—N—N=C, is almost linear, with the C—C—N—N and C—N—N=C torsion angles being −174.1 (2) and 176.0 (2)°, respectively. The dihedral angle between the *p*-toluene­sulfonyl ring and the S—N—C—C(=O) segment is 67.5 (4)°, while that between the two aromatic rings is 52.17 (11)°. In the crystal, the water H atom is involved in O—H⋯O hydrogen bonds with a sulfonamide O atom and the carbonyl O atom. The water O atom is itself hydrogen bonded to both NH hydrogen atoms. These four hydrogen bonds lead to the formation of corrugated sheets lying parallel to (100). There are also weak C—H⋯O contacts present within the sheets.

## Abstract 2

The title aryl­sulfonyl glycinyl hydrazone Schiff base compound crystallizes as a monohydrate. In the crystal, a series of O—H⋯O and N—H⋯O hydrogen bonds leads to the formation of corrugated sheets lying parallel to (100).

## Chemical context   

Hydrazones are an important class of organic compounds in the Schiff base family. The latter display various biological activities such as anti­oxidant, anti-inflammatory, anti­convulsant, analgesic, anti­cancer, anti­parasitic, cardioprotective, anti­depressant, anti­tubercular and anti-HIV activities. The hydrazone Schiff bases of aroyl, acyl, and heteroaroyl compounds are more versatile and flexible due to the presence of the C=O group, an additional donor site. *N*-Acyl­hydrazones containing a glycine residue have been investigated extensively in recent years for their biological and medical activities (Tian *et al.*, 2011 ▸). Acyl­hydrazone derivatives which contain an amino acid moiety and an electron-donating substituent in the sulfonyl phenyl ring have been demonstrated to possess good anti­viral activity (Tian *et al.*, 2009 ▸).
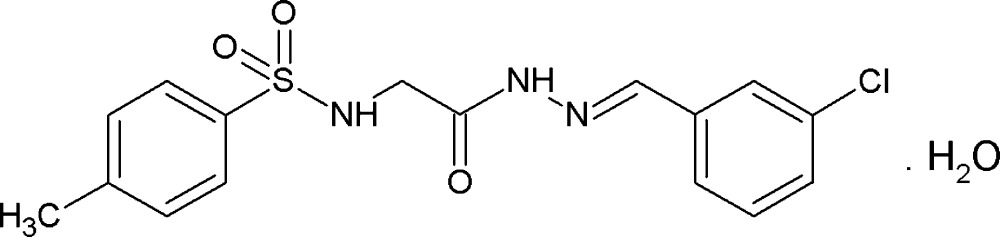


In view of the biological activities of these Schiff bases, which are related to structural aspects, and as part of our studies on the effects of substituents on the structures of *N*-(ar­yl)-amides (Gowda *et al.*, 2000 ▸; Rodrigues *et al.*, 2011 ▸), *N*-chloro­aryl­amides (Jyothi & Gowda, 2004 ▸) and *N*-bromo­aryl-sulfonamides (Usha & Gowda, 2006 ▸), we report herein on the synthesis and crystal structure of the title compound. This acyl­hydrazone derivative contains an amino acid moiety and an electron-donating substituent in the *p*-toluene­sulfonyl ring.

## Structural commentary   

**Figure 1:**
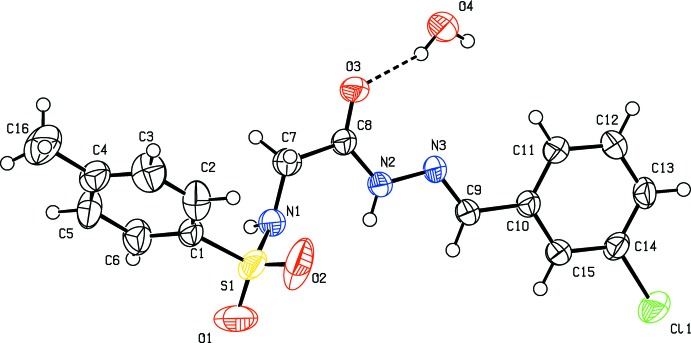
Mol­ecular structure of the title compound, showing the atom labelling. Displacement ellipsoids are drawn at the 50% probability level.

The mol­ecular structure of the title compound is illustrated in Fig. 1 ▸. The conformations of the N—H and C—H bonds in the hydazone part are *syn* to each other, while the N—H and C=O bonds in the central part and the sulfonamide N—H and C—H bonds in the glycine segment are *anti* to each other. The C8—O3 bond length of 1.222 (3) Å indicates that the mol­ecule exists in the keto form in the solid state. The C9—N3 bond length of 1.266 (3) Å confirms its significant double-bond character. The N2—N3 and C8—N2 bond distances are 1.384 (3) and 1.337 (3) Å, respectively, which indicate significant delocalization of the π-electron density over the hydrazone portion of the mol­ecule. The mol­ecule is bent at the S-atom with a S1—N1—C7—C8 torsion angle of 132.0 (2)°. The other central part of the mol­ecule is almost linear with the C7—C8—N2—N3, C8—N2—N3—C9 and N2—N3—C9—C10 torsion angles being −174.1 (2), 176.0 (2) and −176.7 (2)°, respectively. The orientation of the sulfonamide group with respect to the attached *p*-toluene­sulfonyl ring (C1–C6) is given by torsion angles C2—C1—S1—N1 = −99.8 (2)° and C6—C1—S1—N1 = 76.6 (2)°, while that of the hydrazone group with the attached benzene ring (C10-C15) is given by torsion angles C11—C10—C9—N3 = 9.9 (4)° and C15—C10—C9—N3 = −172.1 (2)°. The dihedral angles between the mean plane of the central segment [O3/N2/N3/C7–C9; maximum deviation = 0.065 (3) Å for atom N2] and the benzene rings (C1–C6 and C10–C15) are 65.22 (15) and 13.06 (14)°, respectively. The two benzene rings are inclined to one another by 52.16 (14)°.

## Supra­molecular features   

**Figure 2:**
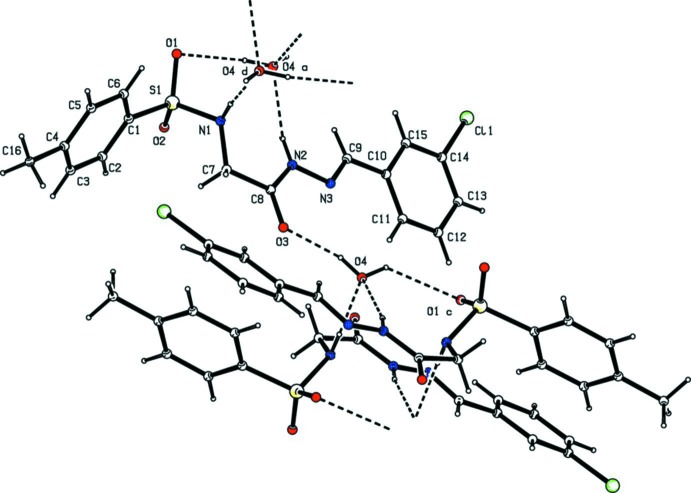
Hydrogen bonding pattern in the title compound [see Table 1 ▸ for details; symmetry codes: (a) −*x* + 1, *y* − 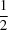, −*z* + 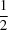; (c) −*x* + 1, *y* + 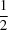, −*z* + 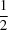; (d) *x*, −*y* + 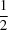, *z* + 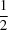].

**Figure 3:**
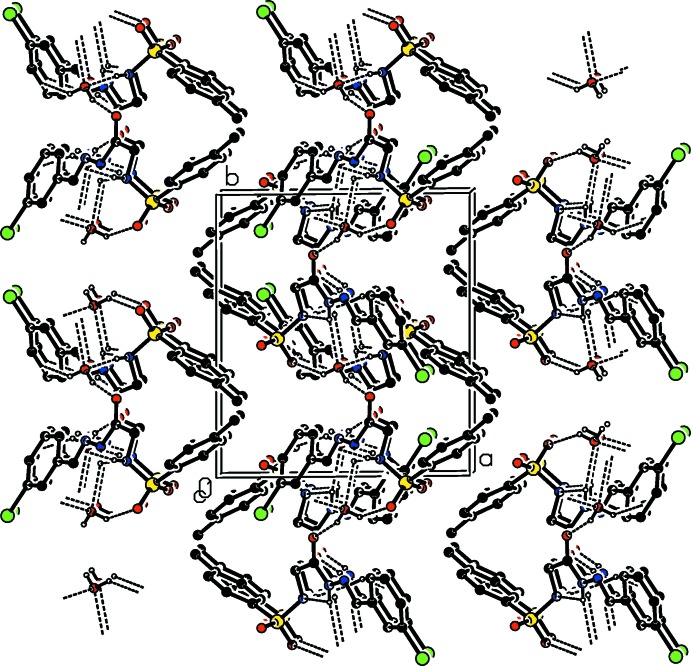
A view along the *c* axis of the crystal packing of the title compound. Hydrogen bonds are shown as dashed lines (see Table 1 ▸ for details), and C-bound H atoms have been omitted for clarity.

In the crystal, the water O-atom, O4, shows bifurcated hydrogen bonding with the amino-H atom of the hydrazide segment (N2) and the sulfonamide-H atom (N1); see Table 1 ▸ and Fig. 2 ▸. One of the H atoms of the water mol­ecule is hydrogen bonded with a sulfonyl O atom, O1, generating 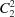
(6) and 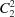
(7) chains. The other H atom shows hydrogen bonding with the carbonyl O atom, O3. These four hydrogen bonds lead to the formation of corrugated sheets lying parallel to (100); see Table 1 ▸ and Fig. 3 ▸. There are also weak C—H⋯O contacts present within the sheets (Table 1 ▸).

## Database survey   

A search of the Cambridge Structural Database (Version 5.36; Groom & Allen, 2014 ▸) for the fragment, *viz.* –NH–CH_2_–C(=O)–NH–N=CH–, yielded only one hit, namely *N*-(2-hy­droxy-1-naphthyl­methyl­ene)-*N*′-(*N*-phenyl­glyc­yl)hydrazine (MEMTOO; Gudasi *et al.*, 2006 ▸).

## Synthesis and crystallization   

The title compound was synthesized in a number of steps. Firstly *p*-toluene­sulfonyl chloride (0.01 mol) was added to glycine (0.02 mol) dissolved in an aqueous solution of potassium carbonate (0.06 mol, 50 ml). The reaction mixture was stirred at 373 K for 6 h, then left overnight at room temperature, filtered and then treated with dilute hydro­chloric acid. The solid *N*-(4-methyl­benzene­sulfon­yl)glycine (**L1**) obtained was crystallized from aqueous ethanol.

Sulfuric acid (0.5 ml) was added to **L1** (0.02 mol) dissolved in ethanol (30 ml) and the mixture was refluxed. The reaction was monitored by TLC at regular inter­vals. After completion of the reaction, the reaction mixture was concentrated to remove excess ethanol. The product, *N*-(4-methyl­benzene­sulfon­yl)glycine ethyl ester (**L2**) obtained was poured into water, neutralized with sodium bicarbonate and recrystallized from acetone.

The pure **L2** (0.01 mol) was then added in small portions to a stirred solution of 99% hydrazine hydrate (10 ml) in 30 ml ethanol and the mixture was refluxed for 6 h. After cooling to room temperature, the resulting precipitate was filtered, washed with cold water and dried to obtain *N*-(4-methyl­benzene­sulfon­yl)glycinyl hydrazide (**L3**).

A mixture of **L3** (0.01 mol) and 3-chloro­benzaldehyde (0.01 mol) in anhydrous methanol (30 ml) and two drops of glacial acetic acid was refluxed for 8 h. After cooling, the precipitate was collected by vacuum filtration, washed with cold methanol and dried. It was recrystallized to constant melting point from methanol (457–458 K). The purity of the title compound was checked and characterized by its IR spectrum. The characteristic absorptions observed are 3253.9, 1680.0, 1597.1, 1334.7 and 1161.2 cm^−1^ for the stretching bands of N—H, C—O, C—N, S—O asymmetric and S—O symmetric, respectively.

Prism-like colourless single crystals of the title compound were grown from a DMF solution by slow evaporation of the solvent.

## Refinement   

Crystal data, data collection and structure refinement details are summarized in Table 2 ▸. The water H atoms were located in a difference Fourier map and refined with the O—H distances restrained to 0.85 (2) Å, and with *U*_iso_(H) = 1.5*U*_eq_(O). The *U*_eq_ of atoms O1 and O2 were restrained to approximate isotropic behaviour. The NH H atoms were also located in a difference Fourier map and refined with U_iso_(H) = 1.2*U*_eq_(N). The C-bound H atoms were positioned with idealized geometry and refined using a riding model: C—H = 0.93–0.97 Å with *U*_iso_(H) = 1.5*U*_eq_(C) for methyl H atoms and 1.2*U*_eq_(C) for other H atoms.

## Supplementary Material

Crystal structure: contains datablock(s) I, global. DOI: 10.1107/S2056989015008506/su5128sup1.cif

Structure factors: contains datablock(s) I. DOI: 10.1107/S2056989015008506/su5128Isup2.hkl

Supporting information file. DOI: 10.1107/S2056989015008506/su5128Isup3.cml

CCDC reference: 1062518

Additional supporting information:  crystallographic information; 3D view; checkCIF report

## Figures and Tables

**Table 1:** Hydrogen-bond geometry (, )

*D*H*A*	*D*H	H*A*	*D* *A*	*D*H*A*
O4H41O3	0.85(3)	1.94(3)	2.752(3)	159(3)
O4H42O1^i^	0.85(3)	2.60(3)	3.274(3)	138(3)
N1H1*N*O4^ii^	0.84(3)	2.06(3)	2.895(4)	171(3)
N2H2*N*O4^iii^	0.84(2)	2.29(2)	3.107(3)	167(2)
C13H13O2^iv^	0.93	2.47	3.366(3)	161
C15H15O3^iii^	0.93	2.59	3.450(3)	155

Symmetry codes: (i) 
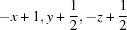
; (ii) 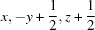; (iii) 
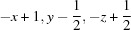
; (iv) 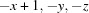.

**Table 2:** Experimental details

Crystal data	
Chemical formula	C_16_H_16_ClN_3_O_3_SH_2_O
*M*_r_	383.84
Crystal system, space group	Monoclinic, *P*2_1_/*c*
Temperature (K)	293
*a*, *b*, *c* ()	12.576(1), 12.769(2), 12.481(1)
()	115.58(1)
*V* (^3^)	1807.8(3)
*Z*	4
Radiation type	Mo *K*
(mm^1^)	0.35
Crystal size (mm)	0.48 0.40 0.36

Data collection	
Diffractometer	Oxford Diffraction Xcalibur Sapphire CCD detector
Absorption correction	Multi-scan (*CrysAlis RED*; Oxford Diffraction, 2009 ▸)
*T*_min_, *T*_max_	0.849, 0.884
No. of measured, independent and observed [*I* > 2(*I*)] reflections	11031, 3307, 2408
*R*_int_	0.026
(sin /)_max_ (^1^)	0.602

Refinement	
*R*[*F* ^2^ > 2(*F* ^2^)], *wR*(*F* ^2^), *S*	0.041, 0.106, 1.04
No. of reflections	3307
No. of parameters	239
No. of restraints	17
H-atom treatment	H atoms treated by a mixture of independent and constrained refinement
_max_, _min_ (e ^3^)	0.24, 0.29

Computer programs: *CrysAlis CCD* and *CrysAlis RED* (Oxford Diffraction, 2009 ▸), *SHELXS97* and *SHELXL97* (Sheldrick, 2008 ▸) and *PLATON* (Spek, 2009 ▸).

## supplementary crystallographic information

### Crystal data

**Table:**

C_16_H_16_ClN_3_O_3_S·H_2_O	*F*(000) = 800
*M**_r_* = 383.84	*D*_x_ = 1.410 Mg m^−^^3^
Monoclinic, *P*2_1_/*c*	Mo *K*α radiation, λ = 0.71073 Å
Hall symbol: -P 2ybc	Cell parameters from 3002 reflections
*a* = 12.576 (1) Å	θ = 3.1–27.8°
*b* = 12.769 (2) Å	µ = 0.35 mm^−^^1^
*c* = 12.481 (1) Å	*T* = 293 K
β = 115.58 (1)°	Prism, colourless
*V* = 1807.8 (3) Å^3^	0.48 × 0.40 × 0.36 mm
*Z* = 4	

### Data collection

**Table:**

Oxford Diffraction Xcalibur Sapphire CCD detector diffractometer	3307 independent reflections
Radiation source: fine-focus sealed tube	2408 reflections with *I* > 2σ(*I*)
Graphite monochromator	*R*_int_ = 0.026
ω scans	θ_max_ = 25.4°, θ_min_ = 3.2°
Absorption correction: multi-scan (*CrysAlis RED*; Oxford Diffraction, 2009)	*h* = −11→15
*T*_min_ = 0.849, *T*_max_ = 0.884	*k* = −14→15
11031 measured reflections	*l* = −14→15

### Refinement

**Table:**

Refinement on *F*^2^	Primary atom site location: structure-invariant direct methods
Least-squares matrix: full	Secondary atom site location: difference Fourier map
*R*[*F*^2^ > 2σ(*F*^2^)] = 0.041	Hydrogen site location: inferred from neighbouring sites
*wR*(*F*^2^) = 0.106	H atoms treated by a mixture of independent and constrained refinement
*S* = 1.03	*w* = 1/[σ^2^(*F*_o_^2^) + (0.0426*P*)^2^ + 0.8977*P*] where *P* = (*F*_o_^2^ + 2*F*_c_^2^)/3
3307 reflections	(Δ/σ)_max_ < 0.001
239 parameters	Δρ_max_ = 0.24 e Å^−^^3^
17 restraints	Δρ_min_ = −0.29 e Å^−^^3^

### Special details

**Table:**

Geometry. All e.s.d.'s (except the e.s.d. in the dihedral angle between two l.s. planes) are estimated using the full covariance matrix. The cell e.s.d.'s are taken into account individually in the estimation of e.s.d.'s in distances, angles and torsion angles; correlations between e.s.d.'s in cell parameters are only used when they are defined by crystal symmetry. An approximate (isotropic) treatment of cell e.s.d.'s is used for estimating e.s.d.'s involving l.s. planes.
Refinement. Refinement of *F*^2^ against ALL reflections. The weighted *R*-factor *wR* and goodness of fit *S* are based on *F*^2^, conventional *R*-factors *R* are based on *F*, with *F* set to zero for negative *F*^2^. The threshold expression of *F*^2^ > σ(*F*^2^) is used only for calculating *R*-factors(gt) *etc*. and is not relevant to the choice of reflections for refinement. *R*-factors based on *F*^2^ are statistically about twice as large as those based on *F*, and *R*- factors based on ALL data will be even larger.

### Fractional atomic coordinates and isotropic or equivalent isotropic displacement parameters (Å^2^)

**Table:**

	*x*	*y*	*z*	*U*_iso_*/*U*_eq_	
Cl1	0.17126 (8)	−0.13298 (6)	−0.26071 (6)	0.0738 (3)	
S1	0.76144 (8)	−0.01311 (5)	0.61570 (6)	0.0673 (3)	
O1	0.7033 (2)	−0.09611 (16)	0.6464 (2)	0.1062 (9)	
O2	0.8221 (3)	−0.0340 (2)	0.54459 (19)	0.1084 (10)	
O3	0.61344 (16)	0.28736 (12)	0.34493 (15)	0.0527 (5)	
N1	0.6578 (2)	0.06969 (17)	0.54323 (18)	0.0531 (6)	
H1N	0.609 (2)	0.075 (2)	0.572 (3)	0.064*	
N2	0.55088 (19)	0.11910 (15)	0.30730 (17)	0.0429 (5)	
H2N	0.550 (2)	0.0597 (15)	0.335 (2)	0.052*	
N3	0.48871 (18)	0.13740 (15)	0.18666 (16)	0.0411 (5)	
C1	0.8618 (2)	0.04984 (19)	0.7459 (2)	0.0455 (6)	
C2	0.9647 (3)	0.0911 (3)	0.7515 (3)	0.0625 (8)	
H2	0.9845	0.0822	0.6884	0.075*	
C3	1.0386 (3)	0.1459 (3)	0.8509 (3)	0.0679 (8)	
H3	1.1078	0.1745	0.8538	0.081*	
C4	1.0120 (3)	0.1590 (2)	0.9455 (2)	0.0558 (7)	
C5	0.9090 (3)	0.1164 (2)	0.9388 (2)	0.0642 (8)	
H5	0.8899	0.1244	1.0025	0.077*	
C6	0.8329 (3)	0.0622 (2)	0.8393 (2)	0.0598 (8)	
H6	0.7631	0.0344	0.8358	0.072*	
C7	0.6874 (3)	0.1691 (2)	0.5065 (2)	0.0592 (8)	
H7A	0.7692	0.1669	0.5196	0.071*	
H7B	0.6800	0.2240	0.5566	0.071*	
C8	0.6120 (2)	0.19752 (18)	0.3780 (2)	0.0404 (6)	
C9	0.4390 (2)	0.05767 (18)	0.12515 (19)	0.0397 (6)	
H9	0.4432	−0.0058	0.1634	0.048*	
C10	0.3749 (2)	0.06248 (17)	−0.00476 (19)	0.0373 (5)	
C11	0.3796 (2)	0.14931 (18)	−0.0694 (2)	0.0437 (6)	
H11	0.4218	0.2083	−0.0301	0.052*	
C12	0.3216 (2)	0.1485 (2)	−0.1919 (2)	0.0485 (6)	
H12	0.3257	0.2068	−0.2346	0.058*	
C13	0.2575 (2)	0.0619 (2)	−0.2517 (2)	0.0474 (6)	
H13	0.2185	0.0614	−0.3343	0.057*	
C14	0.2526 (2)	−0.02361 (18)	−0.1869 (2)	0.0440 (6)	
C15	0.3108 (2)	−0.02469 (18)	−0.0641 (2)	0.0415 (6)	
H15	0.3070	−0.0834	−0.0217	0.050*	
C16	1.0936 (3)	0.2202 (3)	1.0535 (3)	0.0944 (12)	
H16A	1.1740	0.2051	1.0699	0.142*	
H16B	1.0796	0.2005	1.1206	0.142*	
H16C	1.0790	0.2937	1.0385	0.142*	
O4	0.4852 (2)	0.38922 (15)	0.13369 (18)	0.0643 (6)	
H41	0.516 (3)	0.344 (2)	0.189 (3)	0.096*	
H42	0.425 (2)	0.364 (3)	0.077 (3)	0.096*	

### Atomic displacement parameters (Å^2^)

**Table:**

	*U*^11^	*U*^22^	*U*^33^	*U*^12^	*U*^13^	*U*^23^
Cl1	0.0936 (6)	0.0521 (4)	0.0468 (4)	−0.0165 (4)	0.0030 (4)	−0.0128 (3)
S1	0.0968 (6)	0.0392 (4)	0.0341 (4)	0.0075 (4)	−0.0016 (4)	−0.0071 (3)
O1	0.136 (2)	0.0424 (11)	0.0708 (14)	−0.0240 (11)	−0.0207 (13)	0.0124 (10)
O2	0.132 (2)	0.117 (2)	0.0522 (13)	0.0494 (17)	0.0169 (14)	−0.0300 (13)
O3	0.0696 (12)	0.0340 (9)	0.0400 (10)	−0.0012 (8)	0.0100 (9)	0.0021 (7)
N1	0.0679 (16)	0.0407 (12)	0.0300 (11)	−0.0059 (11)	0.0014 (10)	0.0019 (9)
N2	0.0535 (13)	0.0349 (11)	0.0267 (10)	−0.0036 (9)	0.0044 (9)	0.0030 (8)
N3	0.0498 (12)	0.0383 (11)	0.0253 (10)	0.0000 (9)	0.0068 (9)	0.0012 (8)
C1	0.0573 (17)	0.0396 (13)	0.0295 (12)	0.0061 (12)	0.0092 (12)	−0.0005 (10)
C2	0.0617 (19)	0.082 (2)	0.0438 (16)	0.0111 (16)	0.0229 (15)	−0.0023 (14)
C3	0.0481 (17)	0.087 (2)	0.0607 (19)	−0.0048 (16)	0.0166 (15)	0.0003 (17)
C4	0.0548 (18)	0.0494 (15)	0.0440 (16)	0.0016 (13)	0.0032 (13)	−0.0043 (12)
C5	0.073 (2)	0.082 (2)	0.0377 (15)	−0.0082 (17)	0.0235 (15)	−0.0154 (14)
C6	0.0578 (18)	0.0744 (19)	0.0430 (15)	−0.0159 (15)	0.0179 (14)	−0.0065 (14)
C7	0.075 (2)	0.0436 (14)	0.0333 (14)	−0.0112 (13)	−0.0013 (13)	0.0017 (11)
C8	0.0464 (14)	0.0353 (13)	0.0310 (12)	0.0009 (11)	0.0088 (11)	−0.0005 (10)
C9	0.0461 (14)	0.0368 (12)	0.0285 (12)	0.0009 (11)	0.0088 (11)	0.0039 (10)
C10	0.0402 (13)	0.0364 (12)	0.0293 (12)	0.0029 (10)	0.0092 (10)	−0.0002 (9)
C11	0.0521 (15)	0.0377 (13)	0.0364 (13)	−0.0043 (11)	0.0143 (11)	−0.0029 (10)
C12	0.0609 (17)	0.0452 (14)	0.0346 (13)	0.0018 (12)	0.0160 (12)	0.0074 (10)
C13	0.0575 (16)	0.0508 (15)	0.0258 (12)	0.0086 (13)	0.0102 (11)	0.0011 (11)
C14	0.0494 (15)	0.0374 (12)	0.0336 (12)	0.0015 (11)	0.0069 (11)	−0.0073 (10)
C15	0.0492 (15)	0.0351 (12)	0.0339 (12)	0.0009 (11)	0.0120 (11)	0.0021 (10)
C16	0.097 (3)	0.082 (2)	0.065 (2)	−0.019 (2)	−0.0017 (19)	−0.0223 (18)
O4	0.0862 (16)	0.0459 (11)	0.0450 (12)	0.0082 (10)	0.0134 (11)	0.0081 (9)

### Geometric parameters (Å, º)

**Table:**

Cl1—C14	1.741 (2)	C6—H6	0.9300
S1—O2	1.423 (3)	C7—C8	1.512 (3)
S1—O1	1.430 (3)	C7—H7A	0.9700
S1—N1	1.618 (2)	C7—H7B	0.9700
S1—C1	1.763 (2)	C9—C10	1.468 (3)
O3—C8	1.222 (3)	C9—H9	0.9300
N1—C7	1.452 (3)	C10—C15	1.386 (3)
N1—H1N	0.833 (18)	C10—C11	1.387 (3)
N2—C8	1.337 (3)	C11—C12	1.381 (3)
N2—N3	1.384 (3)	C11—H11	0.9300
N2—H2N	0.836 (18)	C12—C13	1.382 (4)
N3—C9	1.266 (3)	C12—H12	0.9300
C1—C2	1.370 (4)	C13—C14	1.376 (3)
C1—C6	1.373 (4)	C13—H13	0.9300
C2—C3	1.377 (4)	C14—C15	1.385 (3)
C2—H2	0.9300	C15—H15	0.9300
C3—C4	1.369 (4)	C16—H16A	0.9600
C3—H3	0.9300	C16—H16B	0.9600
C4—C5	1.374 (4)	C16—H16C	0.9600
C4—C16	1.512 (4)	O4—H41	0.85 (2)
C5—C6	1.382 (4)	O4—H42	0.844 (19)
C5—H5	0.9300		
			
O2—S1—O1	120.10 (18)	N1—C7—H7B	108.6
O2—S1—N1	107.06 (14)	C8—C7—H7B	108.6
O1—S1—N1	104.62 (15)	H7A—C7—H7B	107.6
O2—S1—C1	107.26 (16)	O3—C8—N2	124.6 (2)
O1—S1—C1	109.67 (13)	O3—C8—C7	119.3 (2)
N1—S1—C1	107.51 (11)	N2—C8—C7	116.0 (2)
C7—N1—S1	119.6 (2)	N3—C9—C10	121.9 (2)
C7—N1—H1N	113 (2)	N3—C9—H9	119.1
S1—N1—H1N	112 (2)	C10—C9—H9	119.1
C8—N2—N3	119.05 (19)	C15—C10—C11	119.5 (2)
C8—N2—H2N	120.8 (18)	C15—C10—C9	118.1 (2)
N3—N2—H2N	120.2 (18)	C11—C10—C9	122.4 (2)
C9—N3—N2	115.02 (19)	C12—C11—C10	120.2 (2)
C2—C1—C6	120.2 (2)	C12—C11—H11	119.9
C2—C1—S1	120.5 (2)	C10—C11—H11	119.9
C6—C1—S1	119.2 (2)	C11—C12—C13	120.7 (2)
C1—C2—C3	119.8 (3)	C11—C12—H12	119.7
C1—C2—H2	120.1	C13—C12—H12	119.7
C3—C2—H2	120.1	C14—C13—C12	118.7 (2)
C4—C3—C2	121.2 (3)	C14—C13—H13	120.6
C4—C3—H3	119.4	C12—C13—H13	120.6
C2—C3—H3	119.4	C13—C14—C15	121.5 (2)
C3—C4—C5	118.3 (3)	C13—C14—Cl1	119.37 (18)
C3—C4—C16	120.5 (3)	C15—C14—Cl1	119.12 (19)
C5—C4—C16	121.2 (3)	C14—C15—C10	119.4 (2)
C4—C5—C6	121.5 (3)	C14—C15—H15	120.3
C4—C5—H5	119.3	C10—C15—H15	120.3
C6—C5—H5	119.3	C4—C16—H16A	109.5
C1—C6—C5	119.1 (3)	C4—C16—H16B	109.5
C1—C6—H6	120.5	H16A—C16—H16B	109.5
C5—C6—H6	120.5	C4—C16—H16C	109.5
N1—C7—C8	114.6 (2)	H16A—C16—H16C	109.5
N1—C7—H7A	108.6	H16B—C16—H16C	109.5
C8—C7—H7A	108.6	H41—O4—H42	110 (3)
			
O2—S1—N1—C7	−56.6 (2)	C4—C5—C6—C1	0.6 (5)
O1—S1—N1—C7	174.9 (2)	S1—N1—C7—C8	132.0 (2)
C1—S1—N1—C7	58.4 (2)	N3—N2—C8—O3	3.4 (4)
C8—N2—N3—C9	176.0 (2)	N3—N2—C8—C7	−174.1 (2)
O2—S1—C1—C2	15.1 (3)	N1—C7—C8—O3	165.3 (3)
O1—S1—C1—C2	147.0 (2)	N1—C7—C8—N2	−17.0 (4)
N1—S1—C1—C2	−99.8 (2)	N2—N3—C9—C10	−176.7 (2)
O2—S1—C1—C6	−168.5 (2)	N3—C9—C10—C15	−172.1 (2)
O1—S1—C1—C6	−36.5 (3)	N3—C9—C10—C11	9.9 (4)
N1—S1—C1—C6	76.6 (2)	C15—C10—C11—C12	−0.6 (4)
C6—C1—C2—C3	−0.5 (4)	C9—C10—C11—C12	177.4 (2)
S1—C1—C2—C3	175.8 (2)	C10—C11—C12—C13	0.6 (4)
C1—C2—C3—C4	0.8 (5)	C11—C12—C13—C14	0.0 (4)
C2—C3—C4—C5	−0.4 (5)	C12—C13—C14—C15	−0.5 (4)
C2—C3—C4—C16	−179.4 (3)	C12—C13—C14—Cl1	179.4 (2)
C3—C4—C5—C6	−0.4 (5)	C13—C14—C15—C10	0.4 (4)
C16—C4—C5—C6	178.7 (3)	Cl1—C14—C15—C10	−179.45 (19)
C2—C1—C6—C5	−0.2 (4)	C11—C10—C15—C14	0.1 (4)
S1—C1—C6—C5	−176.6 (2)	C9—C10—C15—C14	−177.9 (2)

### Hydrogen-bond geometry (Å, º)

**Table:**

*D*—H···*A*	*D*—H	H···*A*	*D*···*A*	*D*—H···*A*
O4—H41···O3	0.85 (3)	1.94 (3)	2.752 (3)	159 (3)
O4—H42···O1^i^	0.85 (3)	2.60 (3)	3.274 (3)	138 (3)
N1—H1*N*···O4^ii^	0.84 (3)	2.06 (3)	2.895 (4)	171 (3)
N2—H2*N*···O4^iii^	0.84 (2)	2.29 (2)	3.107 (3)	167 (2)
C13—H13···O2^iv^	0.93	2.47	3.366 (3)	161
C15—H15···O3^iii^	0.93	2.59	3.450 (3)	155

Symmetry codes: (i) −*x*+1, *y*+1/2, −*z*+1/2; (ii) *x*, −*y*+1/2, *z*+1/2; (iii) −*x*+1, *y*−1/2, −*z*+1/2; (iv) −*x*+1, −*y*, −*z*.
